# Estimation of the Availability and Effect of Some European Agro-Industrial By-Products to Reduce the Carbon Footprint of Sheep and Goat Diets

**DOI:** 10.3390/ani16121789

**Published:** 2026-06-09

**Authors:** Mondina Francesca Lunesu, Maria Francesca Caratzu, Silvia Carta, Marco Farina, Anna Nudda, Gianni Battacone, Giuseppe Pulina, Fabio Correddu

**Affiliations:** Department of Agricultural Sciences, University of Sassari, Viale Italia 39A, 07100 Sassari, Italy; mflunesu@uniss.it (M.F.L.); mariafrancescacaratzu@gmail.com (M.F.C.); scarta2@uniss.it (S.C.); m.farina8@phd.uniss.it (M.F.); anudda@uniss.it (A.N.); battacon@uniss.it (G.B.); fcorreddu@uniss.it (F.C.)

**Keywords:** agro-industrial by-product, carbon footprint, environmental impact, sheep, goat

## Abstract

In this study, we aimed to assess the availability and environmental impact of a selection of widespread agro-industrial by-products in Europe and their potential to reduce the environmental impact of small ruminant diets by replacing conventional feed ingredients. Grape, olive, and tomato pomaces and spent grains of beer were considered. The carbon footprint of products (CFP) was used to quantify the environmental impact of agro-industrial by-products, according to the ISO 14067:2018 standard. The system boundary was defined as gate-to-gate, and 1 kg of dried by-product was chosen as the functional unit (FU). The system included the relevant stages of agro-industrial by-product production, from the process of drying agro-industrial by-products to the treatments carried out in the feed industry (e.g., milling, mixing, and pelleting). The CFPs of grape, olive, and tomato pomaces and spent beer grains were 0.26, 0.22, 0.31, and 0.21 kg CO_2_ equivalents (CO_2_*e*)/FU, respectively. Under the assumptions adopted in this scenario-based assessment, reusing grape, olive, tomato, and brewery industry by-products as partial replacements for conventional feed ingredients in sheep and goat diets reduced diet CFP by an average of 23%, with an estimated mitigation potential of 5.15 Mt CO_2_*e*. These values should be interpreted as model-based estimates rather than as directly generalizable mitigation outcomes, because they depend on the selected system boundary, drying assumptions, emission factors, transport exclusion, and the practical feasibility of the dietary substitutions considered. Overall, the results suggest that the recovery of selected agro-industrial by-products may contribute to reducing the environmental impact of conventional feed ingredients when appropriate preservation, logistics, and diet formulation conditions are met.

## Simple Summary

Animal feed production represents a significant source of environmental impact for the livestock sector. Each year, the agri-food system generates a large amount of by-products that can be used as ingredients in the animals’ diets, reducing the proportion of traditional feed and, consequently, their associated climate impact. Although there are many studies in the literature reporting the use of by-products in animal feeding, there is a lack of information regarding their availability and their environmental impact, and, consequently, the actual contribution they can make toward reducing the environmental impact of feed rations. In this study, we estimated the availability and the environmental impact of some widespread by-products in Europe (grape, olive, and tomato pomaces, and spent grains of beer) and their concrete role in reducing the environmental impact of small ruminant diets. Overall, the results confirm that the recovery of some agro-industrial by-products is a sustainable strategy for reducing the environmental impact of conventional ingredients commonly used in animal feeding.

## 1. Introduction

The agro-food system generates large amounts of secondary biomass in the form of agro-industrial by-products, which originate from the processing of both plant and animal products. These include by-products from sugar production, winemaking, and olive oil processing, as well as residues from the processing of tomatoes, potatoes, carrots, citrus fruits, and apples [1]. Many of these by-products, particularly those derived from wine, olive oil, tomato sauce, and beer processing, are rich in valuable nutrients and therefore represent potential feed resources for animal nutrition [2].

Although previous studies have evaluated the composition, feeding value, and environmental relevance of agro-industrial by-products, integrated European-scale assessments of their availability, nutrient recovery, carbon footprint (CFP), and feed substitution potential in small ruminant diets remain limited. Therefore, the present study should be considered as a scenario-based contribution to this still-developing field rather than as a definitive assessment of the full environmental potential of these resources. At the same time, the management of these materials has become increasingly important because of their potential environmental impact, as current food waste management strategies such as incineration, landfill disposal, and composting are often environmentally unsustainable [3]. Among the available recovery strategies, the use of agro-industrial by-products in animal feed has received considerable scientific attention, particularly because of the presence of bioactive compounds [4]; these molecules, often represented by polyphenols, may improve the quality of animal-derived products and the health of animals (pig, poultry and ruminants), thanks to their biological activities as, antimicrobic, anti-inflammatory, antioxidative and cytoprotective effects [5,6,7]. Interest in these agro-industrial by-products is also driven by their chemical composition, offering substantial amounts of protein and fibre. Although several agro-industrial by-products can be included in the diets of both monogastric animals and ruminants, many of these materials are particularly suitable for ruminants because their high fibre content can be efficiently utilized through microbial fermentation in the rumen. Numerous studies have explored their potential inclusion in small ruminant diets, focusing on their effects on milk yield, milk composition, and animal performance [6,7,8,9]. Additional research has examined the impact of grape, olive oil, and brewery by-products on ruminal metabolism in sheep [10,11]. For instance, brewery by-products have been evaluated as alternative protein sources in lamb feeding [12,13,14], though challenges remain regarding the utilization of some by-products, including their high moisture content, which complicates storage and affects microbiological stability. To address these limitations, effective preservation methods, such as drying and ensiling, have been proposed to ensure year-round availability and resolve issues related to the seasonality of production [15,16,17].

The incorporation of food chain by-products into animal diets appears to reduce the environmental impact associated with off-farm feeds, which account for 20% and 23% of total emissions from dairy sheep and dairy goat farms, respectively [18,19]. Moreover, they can directly reduce greenhouse gas (GHG) emissions through their richness in methane-suppressive phenolic compounds [20,21,22], or through reduced disposal, thus supporting circular economy strategies [23]. However, little research has quantified their environmental impact in terms of reducing the environmental footprint of feed in livestock farms. From a life cycle assessment (LCA) perspective, the environmental burden assigned to by-products depends on the allocation method adopted, and this remains a debated issue in the literature. In many studies, by-products are often considered to have negligible GHG emissions, as the environmental burden is typically attributed entirely to the primary product [24]. However, it is necessary to consider post-harvest emissions from the gate of agri-food industries, related to transport, drying, and passages involved in feed chains.

The first objective of this work was to estimate the European availability of nutrients in the valorisation of grape, olive, and tomato pomaces, and brewers’ spent grains. Another objective was to assess the CFP of dried agro-industrial by-products and to evaluate the mitigation potential of including them in the diets of small ruminants.

## 2. Materials and Methods

### 2.1. Chemical Composition Assessment of Agro-Industrial By-Products

A literature review was carried out to assess the chemical composition of selected agro-industrial by-products (grape, olive, and tomato pomaces, and brewers’ spent grains), focusing on the initial contents of moisture, protein, fat, ash, and fibre, expressed as neutral detergent fibre (NDF) and acid detergent fibre (ADF).

Literature data were included when studies reported the chemical composition of the considered by-products on a dry matter (DM) basis, or when values could be reliably converted to DM units. Studies were also required to provide sufficient information to identify the by-product type and processing of origin. When multiple compositional values were available for the same by-product category, data were harmonized to a common DM basis and summarized as mean, standard deviation, minimum, and maximum values. Studies reporting incomplete analytical information, unclear by-product classification, or values that could not be consistently converted to the units adopted in the present work were excluded from the calculation of average composition. This approach was adopted to improve comparability across heterogeneous literature sources, while acknowledging that by-product composition may vary substantially according to raw material characteristics, industrial processing technology, geographical origin, and preservation method. The percentage of by-product obtained from industrial processing was estimated from average literature values. Since these coefficients may vary according to processing technology, raw material characteristics, and regional production systems, the resulting estimates should be interpreted as approximate regional-scale indicators rather than exact quantities.

### 2.2. Availability Estimation of Agro-Industrial By-Products

The amount of available agro-industrial by-products was estimated considering the total production of the primary product in Europe, and the relative percentage of by-products from industrial processing, obtained from the literature. For winery by-products, the quantity of grapes processed into wine was considered. Similarly, the quantity of olive by-products was estimated based on the amount of olives processed for olive production, and tomato by-products were quantified from the amount of tomatoes processed. Brewer’s spent grains were estimated based on total beer production from barley.

Data on the total production of primary products were retrieved from the EUROSTAT [25] and FAOSTAT [26] databases, referring to the production of grapes for wine, olives for oil, and tomatoes in 2023 and malted beer in Europe (EU 27) in 2021. For each primary product, the relative percentage of agro-industrial by-products resulting from industrial processing was estimated as the average percentage reported in the literature. Based on the crude protein and fibre contents, the total amounts of protein and fibre available in Europe were estimated.

### 2.3. Carbon Footprint Assessment of Considered By-Products

The environmental impact of the considered agro-industrial by-products was estimated using the CFP approach, according to the ISO 14067:2018 standard [27]. The system boundary was defined as gate-to-gate, while 1 kg of dried by-product was chosen as the functional unit (FU).

The production processes of agro-industrial by-products were included in the system boundary, but their impacts were considered equal to zero, assuming that at this stage the impacts of these processes are 100% attributed to the primary products. This means that each agro-industrial by-product leaves the primary industry without any environmental impact [28,29]. Detailing, in this assessment, a cut-off approach was adopted: the environmental burdens of the primary agro-industrial processes were attributed to the main products, and the by-products were assumed to enter the feed valorisation chain without upstream burdens. This assumption is commonly used in LCA studies dealing with residues and secondary biomass, but it is not the only possible allocation procedure and remains methodologically debated. Alternative approaches, such as economic allocation, mass allocation, or system expansion, would distribute part of the upstream burden to the by-products and could therefore increase their estimated CFP. The choice adopted here is consistent with the objective of estimating the additional environmental burden associated with transforming available by-products into feed ingredients. It does not aim to reallocate the full environmental impacts of the primary food-processing systems. Accordingly, the CFP values and mitigation potentials reported in this study should be interpreted within the allocation framework adopted. Alternative allocation approaches would likely attribute a higher environmental burden to the by-products considered. The system boundary should therefore be interpreted as a gate-to-gate assessment of the additional processing steps required for feed use, mainly drying and incorporation into compound feeds. Transport was not included because distances between agro-industrial plants, drying facilities, feed mills, and farms are highly context-dependent and cannot be represented by a single European average without introducing additional assumptions. This exclusion is a relevant limitation, particularly for high-moisture by-products, for which transport distance and local availability may strongly affect the final environmental balance. However, pragmatic industrial solutions may reduce this limitation, including on-site moisture reduction at processing facilities through the recovery of residual or low-grade thermal energy that would otherwise be dissipated into the environment.

The impact associated with the drying process was estimated considering the energy required as a function of the by-product’s initial moisture content. For by-products lacking direct literature data on drying energy demand, the electricity requirement was estimated from the initial and final moisture contents and from energy requirements reported for comparable by-products. These estimates should be considered simplified engineering approximations rather than plant-specific measurements, because drying efficiency depends on dryer technology, operating conditions, water removal rate, and energy source. The electricity emission factor used in the baseline calculation was applied as a generalized value; therefore, results may vary according to national or regional electricity mixes and to the degree of decarbonisation of the energy supply. The energy required for the drying process is estimated to evaluate the environmental impact of the drying process for each agro-industrial by-product, as summarized in Table 1. A detailed description of all inputs obtained from the literature and used to estimate the electricity requirement is given in the Supplementary Material (Table S1). The emission factor for electricity consumption was 0.23 kg CO_2_ equivalents (CO_2_*e*)/kWh [30], and for processes related to agro-industrial by-product incorporation into animal feed, an emission factor of 0.04 kg CO_2_*e*/kg by-product (on a fresh matter basis, FM) was considered for each by-product [31].

### 2.4. Calculation of Carbon Footprint Reduction Associated with Feed Substitution

To determine the potential level of inclusion for each by-product, a literature search was carried out to identify studies that included the agro-industrial by-products under investigation as replacements for ingredients in sheep and goat diets. From these studies, only those reporting no negative effects associated with the use of by-products on DM intake and milk production were selected. A list of the selected studies is given in Table S2, together with a detailed description of the level of inclusion of each by-product in the diet and the replaced ingredient.

Feeding studies were selected when they tested one of the considered by-products in sheep or goat diets, clearly reported the inclusion level and the ingredient replaced, and did not show adverse effects on DM intake or productive performance under the experimental conditions. Because the selected studies differed in animal category, basal diet, production system, and replacement strategy, the resulting substitution scenarios were used as illustrative model cases rather than as universal feeding recommendations. The selected diets were derived from experimental studies conducted under specific production conditions and should not be considered fully representative of the diversity of commercial sheep and goat production systems currently present across Europe. Comparability among studies was improved by expressing inclusion and replacement levels on a dietary DM basis and by using the same feed emission factor database for the replaced ingredients.

For each study, the CFP reduction associated with feed substitution was estimated by comparing the CFP contribution of the replaced ingredient and that of the tested by-product at their respective dietary inclusion levels.

The emission factors (EF) of replaced ingredients were obtained from the Global Feed LCA Institute database [34] and are listed in Table 2.

### 2.5. Estimation of Carbon Footprint Reduction at Diet Level and Substitution Scenarios

The reduction in CFP due to substitution was estimated by comparing the control and experimental treatment rations and was based on the level of by-product inclusion in the studies: for each study, the reduction (%) of CFP for each individual substituted ingredient was calculated. Then, four studies that reported the typical proportions of components used in sheep and goat diets were selected to precisely estimate the reduction in CFP associated with the use of each by-product, calculated as the difference between the CFP of the treatment diet (which includes the by-product) and the corresponding control diet (without by-product inclusion). These estimates were made considering diets including olive oil and tomato by-products in sheep [9], spent brewer’s yeast in sheep [11], and grape pomaces in goats [35]; olive, tomato, and grape pomace were used in place of sugar beet pulp, whereas spent brewer’s yeast replaced soybean meal. The diets used to estimate the potential reduction in CFP of by-product diets for each species are given in the Supplementary Material (Table S3 for olive by-products, Table S4 for tomato by-products, Table S5 for grape by-products, and Table S6 for brewers’ spent yeast).

### 2.6. Estimation of Avoided Emissions from By-Product Reuse in Small Ruminant Diets

Based on the estimation of the CFP associated with the control diet (without by-product inclusion), and that associated with the treatment diet (including the by-product), we estimated the avoided impact, expressed as kg CO_2_*e*/kg of diet, as follows:

Avoided impact with the treatment diet, kg CO_2_*e*/kg diet = CFP CON diet, kg CO_2_*e*/kg diet − CFP treatment diet, kg CO_2_*e*/kg diet

The avoided emissions per unit of by-product included in the diet were then calculated by considering the level of inclusion of grape, olive, tomato, and brewery by-products reported in the four diets for sheep and goats, as follows:

Avoided impact, kg CO_2_*e*/kg by-product included = Avoided impact with the treatment diet, kg CO_2_*e*/kg diet:Level of inclusion of by-product, kg/kg dietary DM

The total emissions avoided by using the by-products under study were quantified from the amount of available by-product (tons of DM) estimated in Europe, as follows:

Avoided emissions, Megatons CO_2_*e* = Avoided impact, kg CO_2_*e*/kg by-product included × Available by-product, tons DM:1,000,000

### 2.7. Sensitivity Analysis Assessment

To assess the robustness of the CFP estimates, a deterministic one-at-a-time sensitivity analysis was performed on the main parameters identified as most influential in the model, namely the electricity emission factor, drying energy requirement, and feed substitution ratio (see Supplementary Materials). For the CFP of dried by-products, the sensitivity analysis was performed by applying a ±20% variation to the drying-related CFP component, calculated from the electricity required for drying and the electricity emission factor adopted in the baseline model, while keeping the remaining CFP components constant. This approach was adopted because drying represented the main processing stage included within the system boundary and the principal contributor to the estimated CFP of dried by-products. In addition, the sensitivity of diet CFP reduction was assessed by varying the feed substitution ratio by ±20%, in order to evaluate how changes in the practical inclusion or replacement level of by-products could affect the estimated mitigation potential under different feeding scenarios. Results were expressed as ranges around the baseline CFP values, diet CFP reductions, and estimated avoided emissions.

The adopted deterministic one-at-a-time sensitivity analysis explores the influence of selected parameters under fixed model assumptions and therefore should not be interpreted as a full uncertainty propagation framework. A comprehensive uncertainty assessment would require probabilistic characterization of model inputs and stochastic simulation approaches, which were beyond the scope of the present study.

## 3. Results

### 3.1. Chemical Composition of Agro-Industrial By-Products

A detailed overview of the chemical composition of by-products derived from the wine, olive oil, tomato processing, and beer industries is shown in Table 3. The data highlights the heterogeneity in the nutrient profiles of these by-products.

By-products generally exhibit a high moisture content (greater than 70%), apart from olive oil by-products, which show the lowest moisture level at 50%.

Considering the by-products’ nutrient contents, brewers’ spent grains have the highest average protein content (22.8% DM), making them a valuable protein source. Winery and tomato by-products contain moderate levels of protein (11.6% and 16.0% DM, respectively), while olive oil by-products exhibit the lowest average protein content (8.0% DM). Olive oil and tomato by-products contain relatively higher fat levels (10.4% and 10.8% DM, respectively) compared to winery by-products (6.8% DM) and brewers’ spent grains (8.6% DM), which is consistent with the lipid-rich nature of olive and tomato processing residues. Olive oil and tomato by-products are notably fibre-rich, with average NDF values of 61.65% and 58.4% DM, respectively. Winery by-products also have a substantial fibre fraction (44.0% DM), while brewers’ spent grains have a moderate NDF content (49.9% DM) but the lowest ADF content (20.6% DM).

### 3.2. Availability of Agro-Industrial By-Products

The annual production of by-products from key agro-industrial sectors in Europe is reported in Table 4, along with the estimated availability of crude protein and fibre, providing insights into their nutritional contribution to feed systems. From an FM basis, the beer industry generates the largest volume of by-products (9.56 million tons FM), followed by wineries (4.48 million tons FM) and olive oil (3.98 million tons FM) and tomato processing (0.77 million tons FM).

However, when analyzed on a DM basis, the ranking shifts due to variations in moisture content among the by-products. The beer industry and olive oil sector both produce comparable quantities of by-products, with approximately 2 million tons DM each. This similarity highlights the high DM content of olive oil by-products relative to their lower FM production. In contrast, winery by-products account for 1.29 million tons DM, while tomato processing by-products contribute the least, at just 94,809 tons DM.

### 3.3. Carbon Footprint of Considered By-Products

**Figure 1:**
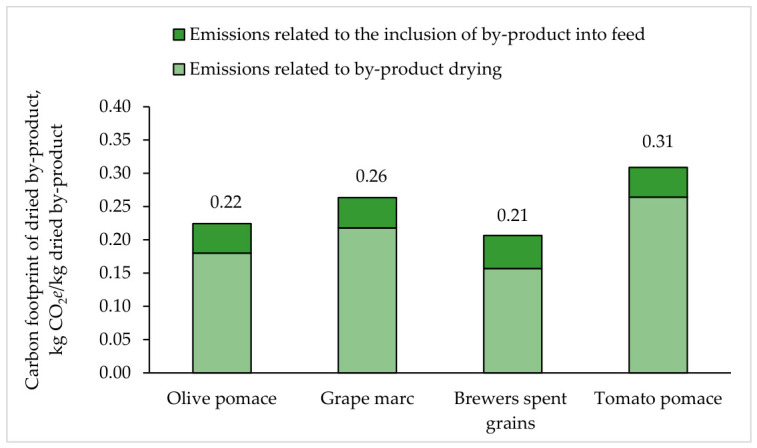
Carbon Footprint (expressed on kg CO_2_*e*/kg dried by-product, on dry matter, DM, basis) of grape, olive, tomato pomace and brewers’ spent grains, estimated by considering the drying process and the processes related to incorporation into animal feed.

The estimated CFPs of the considered by-products are reported in Figure 1; on average, the dried by-products showed an indicative value of 0.25 ± 0.04 (mean ± SD) kg CO_2_*e*/kg (on DM basis), with the lowest values exhibited by brewers’ spent grains and olive pomace (0.21 and 0.22 kg CO_2_*e*/kg, respectively), and the highest value by tomato pomace (0.31 kg CO_2_*e*/kg), while grape pomace showed an intermediate value of 0.26 kg CO_2_*e*/kg. These estimates should be interpreted as indicative values, as they are based on simplified assumptions and a limited system boundary.

### 3.4. Carbon Footprint Reduction Associated with Feed Substitution

The inclusion of 15% and 10% dietary DM grape pomace in sheep and goats, respectively, allowed for a CFP reduction of 43%, related to the use of beet pulp (Table 5).

A reduction of 53% and 55% of the CFP of the concentrate was observed in sheep and goats, respectively, with the inclusion of grape marc. The highest reductions (−76 and −90%) were achieved when grape by-products and brewers spent yeast were used to replace soybean hulls and soybean meal, whose environmental impacts are quite high (1.10 and 2.19 kg CO_2_*e*/kg feed, respectively).

Only one study tested the use of olive cake as a replacement for cotton seed meal [9], showing a 65% reduction in its CFP.

The lowest reductions in CFP of the replaced feed (−19% replacing sugar beet pulp) were observed with the inclusion of tomato pomace at a level of 29.8% in the sheep diet.

### 3.5. Carbon Footprint Reduction at Diet Level and Substitution Scenarios

In this work, the potential reduction in the CFP of the small ruminant diets including by-products was estimated considering the substitution of sugar beet pulp or soybean meal with different by-products, as shown in Table 6. It should be noted that these estimates are highly dependent on the specific control diets and substituted ingredients used as reference scenarios and therefore reflect scenario-specific results rather than general substitution effects. Furthermore, it is clear that the estimated CFP reduction would increase with the amount of by-product included in the diet; however, it should be emphasized that the primary consideration is the nutritional aspect, namely the adverse effects associated with excessive inclusion in the diet. For this reason, the studies used for the estimates, and in particular those employed in this scenario, were selected from among those that reported no adverse effects on animal performance; consequently, the estimates can be considered fairly realistic for a scenario involving the positive utilization of these resources.

The highest CFP reduction (−34.73%) was observed when tomato pomace was used to replace sugar beet pulp, while the lowest CFP reduction (−4.52%) was observed when grape pomace replaced sugar beet pulp. While the replacement of sugar beet pulp with olive pomace allowed for a reduction in the diet’s CFP of −24.01%, the CFP reduction observed in the treatment diet containing brewer’s spent yeast was −28.0%.

### 3.6. Avoided Emissions from By-Product Reuse in Small Ruminant Diets

Table 7 reports the results of the estimated of total CO_2_ emissions avoided, considering the European availability of the four considered by-products, their calculated CFP, and their positive environmental impact when included in the diets. The greatest amount of by-product produced in Europe was that deriving from the beer industry, with more than 2 million tons produced in 2021. This estimate, combined with the highest avoided impact, resulted in 2.0 kg CO_2_*e* avoided per kg of by-product included, associated with the replacement of soybean meal in small ruminant diets; this resulted in the highest contribution to total avoided emissions among the investigated by-products. The total amount of CO_2_ emissions mitigated accounted for 5.15 Mt CO_2_*e*, mostly represented by the Brewers’ spent grains (81%).

### 3.7. Sensitivity Analysis

The sensitivity analysis showed that the CFP estimates of dried by-products were mainly affected by parameters related to the drying process and the electricity emission factor (Table 8). The drying-related CFP component was calculated by multiplying the electricity required for drying by the electricity emission factor adopted in the baseline model. When a ±20% variation was applied to this drying-related component, while keeping the remaining CFP components constant, the CFP ranged from 0.222 to 0.298 kg CO_2_*e*/kg DM for grape pomace, from 0.188 to 0.252 kg CO_2_*e*/kg DM for olive pomace, from 0.263 to 0.357 kg CO_2_*e*/kg DM for tomato pomace, and from 0.184 to 0.236 kg CO_2_*e*/kg DM for brewers’ spent grains. Tomato pomace remained the by-product with the highest CFP under both the low- and high-impact scenarios, confirming the sensitivity of this by-product to drying-related assumptions (Table 8). Sensitivity analysis of the CFP of dried agro-industrial by-products under ±20% variation in energy-related assumptions.

When the feed substitution effect was varied by ±20% (Table 9), the estimated reductions in diet CFP ranged from −3.00% to −4.50% for grape pomace, from −20.00% to −30.00% for olive pomace, from −27.27% to −40.91% for tomato pomace, and from −21.92% to −32.88% for brewers’ spent yeast. These values were calculated by varying the avoided impact between the control and treatment diets while keeping the CFP of the control diet constant.

## 4. Discussion

### 4.1. Chemical Composition and Availability of Agro-Industrial By-Products

High moisture content represents one of the main problems related to the use of agro-industrial by-products at the industry level [100], making by-products quite unstable; to prevent fermentation and oxidation processes, biomass stabilization by rapid drying is needed.

The variability observed in chemical composition (Table 3) reflects the diverse raw materials and processing methods used in different agro-industries. These characteristics underline the potential of these by-products as feed ingredients, albeit with some constraints due to high fibre or moisture contents in certain cases. It should be noted that a large variability in nutrient composition can also be observed within the by-product, due to different industrial processes and geographical contexts. This represents another main limitation related to the widespread and organized use of agro-industrial by-products [23,100].

Analyzing the chemical composition of agro-industrial by-products from a DM-based perspective elucidates the importance of considering moisture content when the potential contribution of agro-industrial by-products to feed systems is evaluated. Importantly, considering the levels of inclusion in the diets (which can be specifical per specie), the availability of these by-products may be more than sufficient to meet the demands of the feed market, not only for small ruminants but also for other livestock species, where they find valuable applications. It should be emphasized that recovering and fully utilizing the calculated quantities of agro-industrial by-products is highly optimistic, given various challenges, particularly logistical and economic ones (difficulties and high costs involved in recovering by-products located far from feed mills and derived from small-scale industrial production). On the other hand, however, it should also be considered that only four by-products were analyzed in this study; many others could help to increase the available quantity of exploitable materials.

Their significant production volumes and nutrient profiles underscore their role in supporting sustainable livestock systems, while also contributing to the circular economy by reducing waste and valorising agro-industrial residues. For these calculations, information regarding the percentage of by-product resulting from the related industrial process is crucial; such information is not easy to obtain, but the data from literature are consistent, as evidenced by the low variability (SD) of these means.

### 4.2. Carbon Footprint of Considered By-Products

To date, little information is present in the literature concerning the quantification of the environmental impact of agro-industrial by-products, making it difficult to compare our results with those of other studies. For some by-products widely used in the feed industry, impact values are quantified by the Global Feed LCA Institute, as is the case for sugar beet pulp, pea and bean hulls, soybean hulls, and oat husk. There is little to no information on the CFP of the by-products considered in this study, despite widespread information on the industry processes of very common products (wine, beer, tomato, and olive oil). From an environmental perspective, our estimated results could be useful for future considerations regarding the use of agro-industrial by-products in the feed industry. The environmental impact of the four by-products was not high compared to the values of typical feed ingredients except for the tomato pomace (0.31 CO_2_*e*/kg DM), the relatively high environmental impact of which can be explained not only by its high moisture content, which ranges from 74.8% to 97.0% [74,96], but also to the higher estimated electricity demand associated with the drying process. This difference may depend on variations in physical structure, water retention capacity, drying efficiency, and process conditions, which can substantially affect the amount of energy required for water removal even among by-products with apparently similar moisture contents. Therefore, moisture content alone cannot fully explain the observed differences in CFP among the considered by-products.

The by-products’ dehydration processes are essential in allowing their further application, due to the high water content of the raw material, which also leads to insufficient biological stability [101,102]. The estimation performed in this work was based on a common drying method for all by-products, although different drying methods may affect the biochemical composition and retention of bioactive compounds, as observed especially in grape pomace [101]. Moreover, the drying process seems to be important in solving the problem related to the seasonality of some productions that results in a high availability of some by-products in a short harvest period [103].

The CFP estimates of by-products observed in this study are in line with environmental impacts associated with by-products such as bean hull and oat husk, which showed CFPs of 0.27 and 0.29 kg CO_2_*e*/kg by-product, respectively [34]. Moreover, according to the GFLI database, wet sugar beet pulp was associated with no environmental impact, while dried beet pulp showed a CFP equal to 0.46 kg CO_2_*e*/kg, suggesting that most of the impact is related to the drying process of the by-product obtained [34]. Lower CFP values were observed for other by-products derived from the milling industry. Rice husk, which is a lignocellulosic agricultural waste product [104], showed a CFP of 0.09 kg CO_2_*e*/kg by-product [34].

It should be noted that the impact related to these by-products’ ensiling techniques (often used as stabilization techniques) and other factors (e.g., transport) should also be considered to provide more accurate results.

Drying is not the only possible stabilization strategy for agro-industrial by-products. Ensiling can be particularly relevant for ruminant systems because it may reduce the need for energy-intensive dehydration and can facilitate local use of high-moisture biomass. However, ensiling also requires appropriate storage infrastructure, control of fermentation quality, and management of seasonal availability. A complete comparison between drying and ensiling would require system-specific data on storage losses, additive use, transport, feed-out management, and animal responses. For this reason, the present drying-based CFP estimates should be considered one possible valorisation pathway rather than a general description of all preservation options.

### 4.3. Carbon Footprint Reduction Associated with Feed Substitution

The wide range (from −19 to −90%) of CFP reductions achieved for the replaced feeds ingredient of the diets could be related to the CFPs of the used by-product, the CFP of the replaced ingredient, the level of inclusion of each by-product, and, finally, the replacement level of substituted feed. The greatest reduction was observed in the replacement of soybean meal with brewers’ spent yeast (−90%), as expected, mainly due to this ingredient having the highest CFP among the replaced feeds considered in this work. The use of olive and tomato pomace to replace cotton seed meal, which had a CFP of 0.63 kg CO_2_*e*/kg, showed quite dissimilar results, even with the same level of by-product inclusion. Besides the different CFP values of the by-products (0.22 and 0.31 kg CO_2_*e*/kg by-product for olive and tomato pomace, respectively) in these diets, olive cake replaced 100% of cotton seed meal, while tomato pomace replaced 75% of the same ingredient.

Most studies considered in this work evaluated the use of wine and olive oil by-products in sheep and goats to replace fibrous concentrates in the diet, such as sugar beet pulp, reaching up to 100% replacement of this ingredient [35,65,99]. Beet pulp is a by-product of the sugar beet industry, widely used as a final feed ingredient due to its high digestible fibre content [105]. Its environmental impact is quite low compared to other substituted feeds considered in this work, as most of the impact is attributed to the primary product of the industrial process. This can explain the intermediate values of CFP reduction observed when grape, olive, and tomato pomace replaced beet pulp in the diets (−43; −52 and −19%, respectively).

The estimated reductions in CFP should be interpreted in the context of the simplified scenarios adopted in this work. The results depend on the composition of the control diets, the CFP of the substituted ingredients, the level of inclusion of each by-product, and the assumption that animal performance is not impaired. In practical conditions, the feasibility of these substitutions also depends on diet balancing, nutrient digestibility, palatability, possible effects of bioactive compounds, preservation method, feed safety, transport distance, and economic convenience. Therefore, the values reported here indicate the potential magnitude of mitigation under defined assumptions, rather than directly transferable reductions for all sheep and goat production systems.

### 4.4. Carbon Footprint Reduction at Diet Level and Substitution Scenarios

The highest reduction in diet CFP was observed with the use of tomato pomace, which was unexpected since it has been shown to have the highest environmental impact of these by-products. It should be noted that in that diet, wheat bran and cotton seed meal were also replaced by tomato pomace, which resulted in a significant reduction in CFP compared to the control diet, as both substituted ingredients have a rather high environmental impact of 0.63 kg CO_2_*e*/kg feed.

Although brewers’ spent yeast had the lowest environmental impact among the four by-products (0.21 kg CO_2_*e*/kg DM of by-product), and it replaced soybean meal, which has a very high CFP (2.19 kg CO_2_*e*/kg feed), the reduction in the diet’s CFP was not the highest, explained by its low level of inclusion (10% of the dietary DM). This is also evident when the results of the grape by-products are observed; indeed, the lowest reduction associated with the use of grape by-products is only related to the lower inclusion level of this by-product in the treatment diet (15% of the dietary DM) compared to the inclusion level of the other by-products. It should be noticed that, in the considered studies, the chosen levels of inclusion of grape pomace were strongly related to its polyphenol and lignin contents. Polyphenols in particular can have very interesting positive effects on the health and productive performance of animals when they are included at low levels in ruminant diets [100] but can have negative impacts on diet digestibility and dry matter intake when included at high levels [35].

### 4.5. Avoided Emissions from By-Product Reuse in Small Ruminant Diets

Tomato industry by-products showed the lowest potential contribution to avoided emissions. This could be related both to the high energy requirements of the drying process, and, therefore, to the highest CFP estimated for the dried by-product, and to the low quantity of this by-product deriving from the primary industry. Indeed, as can be observed in Table 4, for tomato processing, the percentage of by-product arising from agro-industrial processes is on average of 5% (±2.0), the lowest value among those estimated for grape, olive, and brewery by-products. The positive impact on emissions reduction resulting from the use of agro-industrial by-products as animal feed is not insignificant. According to these estimates, under the assumptions adopted in this scenario-based assessment, the use of agro-industrial by-products as animal feed may contribute to reducing the environmental footprint associated with feed production in European livestock systems, especially through the mitigation of emissions related to feed production and utilization. It should be noted that the realistic availability of agro-industrial by-products could be considered optimistic, given various challenges, however, taking into account many other agro-industrial by-products, these estimates should increase, as well as the limitation of emissions linked to the production of raw materials for animal feed.

### 4.6. Sensitivity Analysis

The results of the deterministic one-at-a-time sensitivity analysis confirm that the estimated mitigation potential is sensitive to energy-related assumptions and to the practical level of feed substitution. In particular, the drying step represents a critical point in the valorisation pathway of high-moisture by-products, because changes in drying energy demand or in the electricity emission factor directly affect the CFP assigned to the dried ingredient. This is especially relevant for tomato pomace, whose higher CFP appears to be primarily related not only to moisture content per se, but also to the higher estimated energy demand required for drying. Differences in physical structure, water retention, drying efficiency, and process conditions may therefore explain why by-products with similar moisture content can show different drying-related impacts.

At the diet level, the sensitivity analysis also indicates that the magnitude of CFP reduction depends strongly on the actual replacement level achieved in practice. Therefore, the values reported in this study should be interpreted as scenario-based estimates rather than fixed mitigation coefficients. Furthermore, the deterministic sensitivity analysis performed here was intended to identify the relative influence of key model parameters and not to quantify the overall uncertainty associated with the CFP estimates. Consequently, the reported ranges should be interpreted as indicative sensitivity intervals rather than confidence bounds of the model outputs. Nevertheless, the direction of the effect remained unchanged across the tested ranges, supporting the potential role of selected agro-industrial by-products as circular feed resources when drying, logistics, and diet formulation conditions are favourable.

## 5. Conclusions

The results of this study indicate that, under the assumptions of the present gate-to-gate and scenario-based assessment, selected agro-industrial by-products can contribute to reducing the CFP of small ruminant diets when replacing conventional feed ingredients with higher emission factors. However, this mitigation potential is conditional on the adopted allocation approach, drying energy demand, electricity emission factor, transport logistics, preservation strategy, by-product composition, and the practical feasibility of diet formulation. Therefore, these by-products should be regarded as potentially valuable circular feed resources rather than universally sustainable alternatives, and their implementation should be evaluated within specific local production, processing, and feeding contexts.

Overall, the results of this study suggest that the recovery of selected agro-industrial by-products may represent a potential promising circular strategy for partially reducing the CFP of conventional feed ingredients used in small ruminant diets under specific technological and logistical conditions. Defining appropriate inclusion levels and preservation strategies is essential to support the practical implementation of this approach while maintaining diet quality and animal performance.

The sensitivity analysis confirmed that the estimated CFP values and mitigation potentials are influenced by assumptions related to drying energy demand, electricity emission factors, and practical feed substitution levels. Nevertheless, despite the variability observed under alternative scenarios, the general direction of the results remained unchanged, supporting the potential role of these by-products as circular feed resources for small ruminant systems.

## Figures and Tables

**Table 1:** Initial and final moisture content (%), and electricity required for drying process of grape, olive, and tomato pomace, and brewers’ spent grains.

Agro-Industrial By-Product	Initial Moisture Content (%)	Final Moisture Content (%)	Electricity Required for the Drying Process, kWh/kg By-Product
Grape pomace ^1^	71	12	0.83
Olive pomace ^2^	60	10	0.70
Tomato pomace ^1^	88	10	1.03
Brewers spent grains ^3^	81	20	0.57

^1^ Estimated by authors; ^2^ from Ramos and Ferreira [32]; ^3^ from Ortiz et al. [33].

**Table 2:** Emission factors of the ingredients included in the diets of the considered studies (From GFLI [34]).

Substituted Feed	EF of Replaced Feeds, kg CO_2_*e*/kg Feed
Alfalfa hay	0.15
Barley	0.29
Barley grain	0.53
Barley straw	0.04
Canola meal	0.49
Concentrate	0.68
Corn	0.39
Corn silage	0.56
Cotton seed meal	0.63
Soybean hull	1.10
Soybean meal	2.19
Sugar beet pulp	0.46
Sunflower meal	0.85
Wheat	0.51
Wheat bran	0.63
Wheat straw	0.04

Abbreviations: CO_2_*e* = CO_2_ equivalents; EF = Emission factor.

**Table 3:** Chemical composition of agro-industrial by-products derived from the wine, olive oil, tomato processing, and beer industries. Data are reported as mean, standard deviation (SD), minimum (Min) and maximum (Max).

Item	Winery By-Products ^1^				Olive Oil By-Products ^1^				Tomato By-Product ^1^				Brewers’ Spent Grains ^1^			
	Mean	SD	Min	Max	Mean	SD	Min	Max	Mean	SD	Min	Max	Mean	SD	Min	Max
Moisture, %	71.2	9.5	63.0	81.7	55.0	7.1	50.0	60.0	87.7	13.2	78.4	97	78.1	4.07	70.0	85.0
Dry matter, % as fed	92.3	1.7	88.6	95.0	85.7	8.0	77.5	94.7	92.7	2.5	90.3	95.2	90.0	-	-	-
Protein, % DM	11.6	1.8	8.5	16.3	8.0	0.7	7.2	9.2	16.0	2.6	11.7	19.1	22.8	4.96	14.2	31.0
Fat, % DM	6.8	2.1	3.9	11.4	10.4	5.6	5.4	16.5	10.8	6.0	5.4	22.2	8.6	3.29	4.5	13.0
NDF, % DM	44.0	7.3	32.0	55.8	61.65	2.4	58.4	64.1	58.4	4.5	55.2	61.6	49.9	0.07	49.8	49.9
ADF, % DM	37.2	5.8	29.8	49.2	51.0	3.4	45.9	54.4	48.5	3.2	46.2	50.7	20.6	4.45	17.4	23.7
Ash, % DM	7.0	2.8	2.1	12.1	9.6	5.2	3.7	13.6	4.1	0.7	3.1	4.8	3.2	1.1	1.1	4.6

Abbreviations: DM = dry matter; NDF = neutral detergent fibre; ADF = acid detergent fibre. ^1^ The average values were calculated by the authors. Supported literature: Winery by-products: [6,10,36,37,38,39,40,41,42,43,44,45,46,47,48,49,50,51,52,53,54,55,56,57,58,59,60,61]. Olive oil by-products: [17,39,62,63,64,65,66,67,68,69,70,71]. Tomato by-products: [6,65,72,73,74,75]. Brewers’ spent grains: [76,77,78,79,80,81,82,83,84,85,86,87,88].

**Table 4:** Estimated amount of agro-industrial by-products, crude protein and fibre available in Europe.

Item	Winery By-Products ^1^	Olive Oil By-Products ^1^	Tomato By-Product ^1^	Brewers’ Spent Grains ^1^
Primary product, tons ^1^	21,087,560	9,359,670	15,416,020	50,307,660
By-product from agro-industrial processes, % ^2^	21.3 ± 1.4	42.5 ± 11.7	5.0 ± 2.10	19.0 ± 1.7
Agro-industrial by-product, tons (FM)	4,481,107	3,977,860	770,801	9,558,455
Agro-industrial by-product, tons (DM)	1,289,662	1,790,037	94,809	2,092,240
Available protein, tons	149,878	176,935	15,177	477,703
Available NDF, tons	567,188	1,363,507	55,368	1,042,981

Abbreviations: DM = dry matter; FM = fresh matter; NDF = neutral detergent fibre. ^1^ Data referred to 2023 for winery, olive oil, and tomato by-products, and to 2021 for beer industry by-products. For the winery, olive oil and tomato by-products, the quantity of grapes, olives and tomatoes processed were considered. Brewer’s spent grains were instead estimated based on total beer production from barley. ^2^ The average values and SD were calculated by the authors. Supported literature: Winery by-products: [41,89,90,91]. Olive oil by-products: [62,63,92,93]. Tomato by-products: [94,95,96]. Beer industry by-products: [76,83].

**Table 5:** Estimated reduction (%) of the carbon footprint of different replaced feeds in the diets by the different by-products considered.

References	Species	By-Product	By-Product Inclusion, % of Dietary DM	Substituted Feed	Replacement Level, %	Reduction in the CFP of Replaced Feed, %
[61]	Goat	Grape marc	18.9	Concentrate	24	−55%
[35]	Goat	Grape marc	15.0	Sugar beet pulp	100	−43%
[97]	Goat	Grape seed cake	5.0	Soybean hull	50	−76%
[98]	Goat	Tomato pomace	24.0	Wheat bran	100	−51%
[99]	Sheep	Grape pomace	10.0	Sugar beet pulp	100	−43%
[61]	Sheep	Grape marc	19.6	Concentrate	27	−53%
[9]	Sheep	Olive cake	29.8	Cotton seed meal	100	−65%
[65]	Sheep	Olive cake	30.0	Sugar beet pulp	100	−52%
[9]	Sheep	Tomato pomace	29.8	Sugar beet pulp	83	−19%
[11]	Sheep	Brewers spent yeast	10.0	Soybean meal	100	−90%

Abbreviations: CFP = carbon footprint; DM = dry matter.

**Table 6:** Estimated reduction in the carbon footprint of diets including grape, olive, and tomato pomace by-products replacing the sugar beet pulp or soybean meal compared to the control diet.

Agro-Industrial By-Product	CFP CON Diet, kg CO_2_*e*/kg Diet	CFP Treatment Diet, kg CO_2_*e*/kg Diet	Reduction in the Diet CFP, %	Replaced Feed	Level of Feed Replacement, %
Grape pomace	0.80	0.77	−4.52	Sugar beet pulp	100
Olive pomace	0.44	0.33	−25.01	Sugar beet pulp	100
Tomato pomace	0.44	0.29	−34.73	Sugar beet pulp	83
Brewers’ spent yeast	0.73	0.53	−28.0	Soybean meal	100

Abbreviations: CFP = carbon footprint; CON = control diet.

**Table 7:** Estimation of CO_2_ emissions saved using agro-industrial by-products in small ruminant diets.

Agro-Industrial By-Product	Level of Inclusion of By-Product, kg/kg Dietary DM	Avoided Impact with the Treatment Diet, kg CO_2_*e*/kg Diet	Avoided Impact, kg CO_2_*e*/kg By-Product Included	By-Product Available, Tons DM	Avoided Emissions, Megatons CO_2_*e*
Grape pomace	0.150	0.03	0.20	1,289,662	0.26
Olive pomace	0.298	0.11	0.37	1,790,037	0.66
Tomato pomace	0.298	0.15	0.50	94,809	0.05
Brewers’ spent grains	0.100	0.20	2.00	2,092,240	4.18
Total					5.15

Abbreviations: DM = dry matter.

**Table 8:** Sensitivity analysis performed by applying a ±20% variation to the drying-related carbon footprint component calculated from electricity demand and electricity emission factor.

Agro-Industrial By-Product	Electricity Required for Drying (kWh/kg)	Baseline CFP (kg CO_2_*e*/kg DM)	Drying-Related CFP (kg CO_2_*e*/kg DM)	Sensitivity Range (kg CO_2_*e*/kg DM)
Grape pomace	0.83	0.260	0.191	0.222–0.298
Olive pomace	0.70	0.220	0.161	0.188–0.252
Tomato pomace	1.03	0.310	0.237	0.263–0.357
Brewers’ spent grains	0.57	0.210	0.131	0.184–0.236

Abbreviations: CFP = carbon footprint; DM = dry matter.

**Table 9:** Sensitivity analysis of the reduction in diet carbon footprint under ±20% variation in feed substitution ratio.

Diet Including By-Product	CFP CON Diet (kg CO_2_*e*/kg Diet)	CFP Treatment Diet (kg CO_2_*e*/kg Diet)	Avoided Impact (kg CO_2_*e*/kg Diet)	Baseline Reduction in Diet CFP (%)	Sensitivity Range (%)
Grape pomace	0.80	0.77	0.03	−3.75	−3.00 to −4.50
Olive pomace	0.44	0.33	0.11	−25.00	−20.00 to −30.00
Tomato pomace	0.44	0.29	0.15	−34.09	−27.27 to −40.91
Brewers’ spent yeast	0.73	0.53	0.20	−27.40	−21.92 to −32.88

Abbreviations: CFP = carbon footprint.

## Data Availability

The original contributions presented in this study are included in the article/Supplementary Materials. Further inquiries can be directed to the corresponding author.

## Supplementary Materials

The following supporting information can be downloaded at: https://www.mdpi.com/article/10.3390/ani16121789/s1, Table S1: Input retrieved from literature for estimating the carbon footprint of dried grape, olive, tomato and beer industry by-products. Table S2: List of selected studies from literature considered in this work using grape, olive, tomato pomace and brewers spent grains in the diet of sheep and goats. Table S3: Typical diet considered for the estimation of the carbon footprint of the control diet and the diet including olive oil by-products in sheep. From Abbeddou et al. [9]. Table S4: Typical diet considered for the estimation of the carbon footprint of the control diet and the diet including tomato by-products in sheep. From Abbeddou et al. [9]. Table S5: Typical diet considered for the estimation of the carbon footprint of the control diet and the diet including grape by-products in goats. From Badiee Baghsiyah et al. [35]. Table S6: Typical diet considered for the estimation of the carbon footprint of the control diet and the diet including beer industry by-products in sheep. From Oancea et al. [11].

## Abbreviations

The following abbreviations are used in this manuscript:

ADF	Acid Detergent Fibre
CFP	Carbon Footprint of product
CO_2_*e*	CO_2_ equivalents
DM	Dry Matter
FM	Fresh Matter
FU	Functional unit
GHG	Greenhouse gas
LCA	Life Cycle Assessment
NDF	Neutral Detergent Fibre
