# Supplementary material for: Estimation of the Availability and Effect of Some European Agro-Industrial By-Products to Reduce the Carbon Footprint of Sheep and Goat Diets
Source: Animals (Basel). 2026 Jun 9;16(12):1789. doi: 10.3390/ani16121789 (PMC13295385; doi:10.3390/ani16121789)
Supplement: Supplementary file 1 [file animals-16-01789-s001.zip › animals-4274831-supplementary.pdf]

Supplementary Materials

Table S1. Input retrieved from literature for estimating the carbon footprint of dried grape, olive, tomato and beer industry by-products.

Olive pomace		**References**
Initial moisture content, %	0.60	Ramos and Ferreira, 2022
Final moisture content, %	0.10	Ramos and Ferreira, 2022
Thermal energy (heat) required for the drying process, MJ/kg by-product	2.61	Ramos and Ferreira, 2022
Conversion of MJ into kWh	0.27	
Electricity required for the drying process, kWh/kg by-product	0.70	
Grape pomace (GP)		
Functional unit (FU)	1 ton of GP	Cortés et al., 2020
System boundary	Cradle-to-gate	Cortés et al., 2020
Environmental impact associated to grape pomace production, kg CO_2e/FU	0	Cortés et al., 2020
Environmental impact associated to grape marc production, kg CO_2e/FU	0	Benetto et al., 2015
Initial moisture content, %	0.71	Benetto et al., 2015
Final moisture content, %	0.12	Benetto et al., 2015
Thermal energy (heat) required for the drying process, MJ/kg by-product	3.09	
Conversion of MJ into kWh	0.27	
Electricity required for the drying process, kWh/kg by-product	0.83	
Brewers spent grains (BSG)		
Initial moisture content, %	0.79	Ortiz et al., 2019
Final moisture content, %	0.20	Ortiz et al., 2019
Specific energy consumption, MJ/kg water evaporated	3.42	Ortiz et al., 2019
Conversion of MJ into kWh	0.27	
Electricity required for the drying process, kWh/kg by-product	0.55	
Tomato pomace		
Initial moisture content, %	0.88	
Final moisture content, %	0.10	
Thermal energy required for the drying process, MJ/kg by-product	3.81	
Conversion of MJ into kWh	0.27	
Electricity required for the drying process, kWh/kg by-product	1.03	
Emissions related to the processes of inclusion of by-product in the feed, kg CO_2e/kg by-product [1]	0.04	ASSALZOO, 2020

[1] This coefficient of emission has been applied for all by-products considered.

Table S2. List of selected studies from literature considered in this work using grape, olive, tomato pomace and brewers spent grains in the diet of sheep and goats.

References	Species	Tested by-product	By-product inclusion, % of dietary DM	Substitute feed	Replaced feed in the diet, % of dietary DM	Replacement level, %
Tsiplakou and Zervas 2008	Goat	Grape marc	18.9	Concentrate	16	24
Badiee Baghsiyah et al., 2023	Goat	Grape marc	15.0	Sugar beet pulp	15	100
Antunović et al., 2024	Goat	Grape seed cake	5.0	Soybean hull	5	50
Razzaghi et al., 2015	Goat	Tomato pomace	24.0	Wheat bran	24	100
Bennato et al., 2022	Sheep	Grape pomace	10.0	Sugar beet pulp	10	100
Tsiplakou and Zervas, 2008	Sheep	Grape marc	19.6	Concentrate	16	27
Abbeddou et al., 2015	Sheep	Olive cake	29.8	Cotton seed meal	29.8	100
Abbeddou et al., 2011	Sheep	Olive cake	30.0	Sugar beet pulp	30	100
Chiofalo et al., 2004	Sheep	Olive cake	6.4	Sugar beet pulp	13	100
Chiofalo et al., 2004	Sheep	Olive cake	6.4	Wheat flour	10	100
Abbeddou et al., 2015	Sheep	Tomato pomace	29.8	Sugar beet pulp	24.8	83
Abbeddou et al., 2015	Sheep	Tomato pomace	29.8	Cotton seed meal	14.9	75
Oancea et al., 2023	Sheep	Brewers spent yeast	10.0	Soybean meal	10.0	100

Abbreviations: DM = dry matter.

Table S3. Typical diet considered for the estimation of the carbon footprint of the control diet and the diet including olive oil by-products in sheep. From Abbeddou et al., 2015.

Diet composition, g/kg DM	Control diet	Treatment diet containing dried olive cake
Barley grain	99	298
Molasses	0	99
Mineral- vitamin premix	8	8
Barley straw	298	198
Sugar beet pulp	298	0
Cotton seed meal	198	99
Wheat bran	99	0
Olive cake	0	298
Total	1000	1000

Abbreviations: DM = dry matter.

Table S4. Typical diet considered for the estimation of the carbon footprint of the control diet and the diet including tomato by-products in sheep. From Abbeddou et al., 2015.

Diet composition, g/kg DM	Control diet	Treatment diet containing tomato pomace
Barley grain	99	198
Barley straw	298	298
Sugar beet pulp	298	50
Cotton seed meal	198	49
Wheat bran	99	0
Molasses	0	99
Mineral- vitamin premix	8	8
Tomato pomace	0	298
Total	1000	1000

Abbreviations: DM = dry matter.

Table S5. Typical diet considered for the estimation of the carbon footprint of the control diet and the diet including grape by-products in goats. From Badiee Baghsiyah et al., 2023.

**Diet composition, g/kg DM**	**Control diet**	**Treatment diet containing grape by-products**
Alfalfa hay	120	120
Wheat straw	120	120
Corn silage	200	200
Barley	115	115
Corn	90	90
Soybean meal	70	70
Canola meal	30	30
Wheat bran	80	80
Salt	3	3
Calcium bicarbonate	7	7
Bicarbonate sodium	5	5
Vitamin-mineral premix	10	10
Beet pulp	150	0
Grape by-products	0	150
Total	1000	1000

Abbreviations: DM = dry matter.

Table S6. Typical diet considered for the estimation of the carbon footprint of the control diet and the diet including beer industry by-products in sheep. From Oancea et al., 2023.

**Diet composition, g/kg DM**	**Control diet**	**Treatment diet containing brewers spent yeast**
Maize	500	330
Wheat	230	400
Wheat bran	30	30
Sunflower meal	100	100
Soybean meal	100	0
Brewers spent yeast	0	100
Calcium	20	20
Sodium	10	10
Mineral-vitamin supplement	10	10
Total	1000	1000

Abbreviations: DM = dry matter.

Supplementary Note – Sensitivity Analysis Methodology

The deterministic one-at-a-time sensitivity analysis was performed to evaluate the effect of uncertainty associated with drying-related energy demand and feed substitution assumptions on the estimated carbon footprint (CFP) values and mitigation potentials.

For the CFP of dried agro-industrial by-products, the drying-related CFP component was calculated as:

$$CFP\_drying = Electricity\ required\ for\ drying \times Electricity\ emission\ factor$$

using an electricity emission factor of 0.23 kg CO2e/kWh.

The baseline CFP values reported in the manuscript were separated into drying-related and remaining fixed contributions according to:

$$CFP\_other = CFP\_total - CFP\_drying$$

where CFP_other represents the remaining fixed contribution associated with feed incorporation processes.

A ±20% variation was then applied only to the drying-related CFP component while maintaining the remaining CFP contribution constant:

$$CFP\_low = CFP\_other + 0.8 \times CFP\_drying$$

$$CFP\_high = CFP\_other + 1.2 \times CFP\_drying$$

For the sensitivity analysis of diet CFP reduction, the avoided impact between the control and treatment diets was first calculated as:

$$Avoided\ impact = CFP\_control - CFP\_treatment$$

The percentage reduction in diet CFP was then calculated as:

$$Reduction\ (\%) = (CFP\_treatment - CFP\_control)\ /\ CFP\_control \times 100$$

The corresponding lower- and higher-impact scenarios were obtained by applying ±20% variation to the avoided CFP difference between control and treatment diets:

$$Reduction\_lesser = 0.8 \times Reduction\_baseline$$

$$Reduction\_greater = 1.2 \times Reduction\_baseline$$

For the estimation of avoided emissions at the European scale, the baseline avoided emissions calculated for each by-product were also varied by ±20% according to:

$$Avoided\ emissions\_lesser = 0.8 \times Avoided\ emissions\_baseline$$

$$Avoided\ emissions\_greater = 1.2 \times Avoided\ emissions\_baseline$$

This sensitivity analysis was intended to evaluate the robustness of the estimated mitigation potentials under alternative assumptions related to drying energy demand and practical feed substitution scenarios, rather than to provide a full probabilistic uncertainty analysis.
